# A library of protein surface patches discriminates between native structures and decoys generated by structure prediction servers

**DOI:** 10.1186/1472-6807-11-20

**Published:** 2011-05-04

**Authors:** Roi Gamliel, Klara Kedem, Rachel Kolodny, Chen Keasar

**Affiliations:** 1 Department of Computer Science, Ben-Gurion University of the Negev, Beer-Sheva, Israel; 2 Department of Computer Science, University of Haifa, Haifa, Israel

## Abstract

**Background:**

Protein surfaces serve as an interface with the molecular environment and are thus tightly bound to protein function. On the surface, geometric and chemical complementarity to other molecules provides interaction specificity for ligand binding, docking of bio-macromolecules, and enzymatic catalysis.

As of today, there is no accepted general scheme to represent protein surfaces. Furthermore, most of the research on protein surface focuses on regions of specific interest such as interaction, ligand binding, and docking sites. We present a first step toward a general purpose representation of protein surfaces: a novel surface patch library that represents most surface patches (~98%) in a data set regardless of their functional roles.

**Results:**

Surface patches, in this work, are small fractions of the protein surface. Using a measure of inter-patch distance, we clustered patches extracted from a data set of high quality, non-redundant, proteins. The surface patch library is the collection of all the cluster centroids; thus, each of the data set patches is close to one of the elements in the library.

We demonstrate the biological significance of our method through the ability of the library to capture surface characteristics of native protein structures as opposed to those of decoy sets generated by state-of-the-art protein structure prediction methods. The patches of the decoys are significantly less compatible with the library than their corresponding native structures, allowing us to reliably distinguish native models from models generated by servers. This trend, however, does not extend to the decoys themselves, as their similarity to the native structures does not correlate with compatibility with the library.

**Conclusions:**

We expect that this high-quality, generic surface patch library will add a new perspective to the description of protein structures and improve our ability to predict them. In particular, we expect that it will help improve the prediction of surface features that are apparently neglected by current techniques.

The surface patch libraries are publicly available at http://www.cs.bgu.ac.il/~keasar/patchLibrary.

## Background

Protein surfaces attract numerous studies as they are the site of molecular binding and enzymatic reactivity. To date these studies use three levels of protein surface representations. The oldest represents surfaces as sets of exposed atoms [1]. A common alternative is to represent surfaces by sets of mesh points [2-4] that smooth the exposed atom surfaces. Finally, sets of mesh points may be coarse grained by descriptor-based methods [5-7] that allow rapid comparisons of surfaces and surface patches. These representations have served as an infrastructure for numerous studies that analyze surface electrostatics [8,9], predict catalytic residues and active sites [10], and characterize binding sites for small ligands as well as other proteins (for recent reviews see [7,11,12]). While these studies mark a major trend in the annotation and prediction of protein function, surfaces are practically ignored in protein structure prediction. Specifically, we are not aware of any study that tried to assess the surfaces of models generated by prediction methods. This is somewhat surprising as one of the ultimate goals of structure prediction is to allow functional annotation of the target proteins and to support structure-based design of ligands and mutations [13]. The current study suggests a plausible approach to the assessment of model surfaces and compares surface accuracy with standard backbone-based measures such as Root Mean Square Deviation (RMSD) [14] or Global Distance Test - Total Score [15].

Notwithstanding the importance of the fine-grained representations of protein surfaces, their complexity calls for coarse graining, or abstraction; a coarser perspective can reveal new insights about the surface architecture that are otherwise masked by the plethora of fine details. Two previous lines of study, [16-18], and [19-21], suggested coarse-grained representation of protein surfaces using the notion of surface patches. Their approaches to the problem were remarkably different, reflecting the different aims of these studies. Jones and Thornton [16,17] and later Albou et al. [18] defined surface patches as overlapping sets of proximate surface residues, and compared binding site patches with non-binding ones to characterize and predict protein-protein interaction sites [22]. Baldacci et al. [19,20] defined surface patches as non-overlapping sets of homogeneous and connected surface points and classified them to twelve predefined types. They employed data mining techniques on these patches to identify structural similarity and plausible evolutionary connection between proteins. Since both applications of the surface patch concept are so tightly tailored to their specific aim, it is hard to see how they can be used in a different context.

Here we present a more general representation of surface patches, which is inspired by the central role of clustering in the study of protein fragments (i.e., contiguous structural segments along the protein chain) [23]. Representative fragments, extracted by clustering large data sets of protein structure fragments, have been used for a wide range of applications including: studies of sequence/structure relationships [24,25], sequence alignment [26], structural comparison and classification [27], large scale mapping of the fold space of proteins [28], and for protein structure prediction [26,29]. Here, we use the K-means++ [30] clustering algorithm to generate a library of representative protein surface-patches that commonly occur in the Protein Data Bank (PDB). To demonstrate the utility of our approach, we quantify the differences between the surfaces of native protein structures and those of decoys generated by state-of-the-art structure prediction methods. We also suggest a variety of other applications for future research.

**Figure 1:**
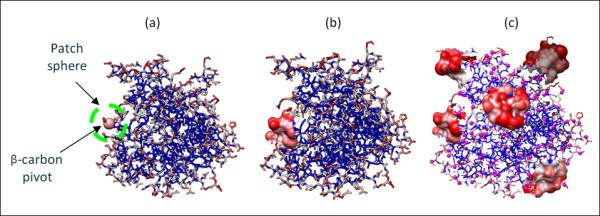
**Illustration of surface patches extracted from the crystal structure of asparagine synthetase (12AS)**. (a) The protein's atoms, color coded by solvent exposure from exposed (red) to buried (blue). A surface patch consists of all the exposed atoms within a sphere of radius *r *= 7Å (green) around a central surface β-carbon (pivot enlarged for illustration). (b) The surface of a single patch. (c) Neighboring patches on the protein surface typically overlap (the small magenta spheres represent pivots).

**Figure 2:**
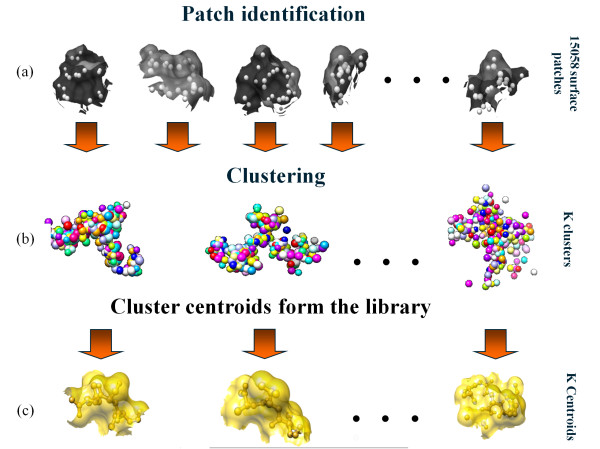
**Construction of the patch library. **(a) First, we extract the surface patches from the data set; atoms are marked by small spheres in the patch. (b) Then, we group of the patches into *k *clusters; the atoms of the patches in each cluster are superimposed on the cluster centroid. For clarity, we omit the surfaces, and render the atoms of each patch in the cluster in a different color. (c) The surface patch library is represented by the cluster centroids.

**Figure 3:**
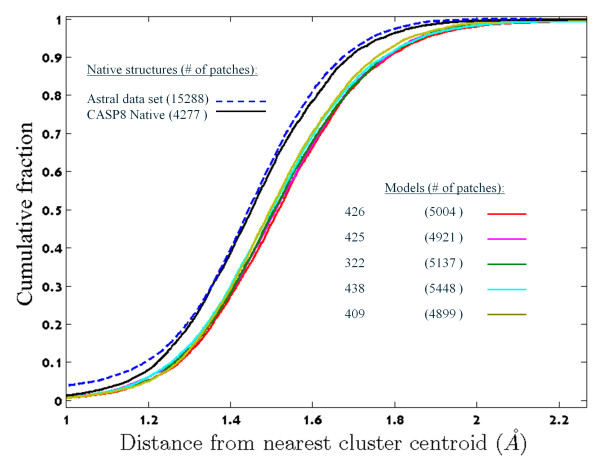
**The cumulative distributions of the distance of native and decoy patches to their closest cluster centroid in the surface patch library**. The fit to the library of patches from native structures (in dashed blue and solid black) is significantly different from that of CASP8 server models (*p*< 10^-42 ^by Wilcoxon rank sum test).

Briefly, a surface patch in this study is a set of surface atoms within a certain radius around a surface β-carbon, denoted the pivot (Figure 1). The distance between two patches is the Root Mean Square Deviation (RMSD) between their atoms under a mapping that preserves chemical identity. Pairs of patches of different chemical compositions are considered infinitely distant. The K-means++ algorithm uses this distance to break a large data set of patches into *k *= 350 structurally homogeneous clusters. The centroids of these clusters constitute our library (Figure 2), which captures genuine features of native structures surfaces (Figure 3).

## Results

We extracted 15,288 surface patches from the training set domains, calculated all vs. all distances, and weeded out 200 outlier patches that were too far from most other patches. Then, using the K-means++ algorithm [30] we divided the patches to *k *= 350 clusters. The algorithm associates each cluster with a representative centroid. The set of 350 centroids constitutes a library of surface patches (Figure 2). Given this library, any surface patch may be associated with the closest library element, and the surface of any protein structure may be described by a list of the associated library elements.

Below, we compare the library-compatibility of the training-set proteins to the compatibilities of the test-set native structures and their decoys. We further compare the compatibilities of the decoys themselves, attempting to correlate it with the decoy quality.

### Distribution of native and decoy patch distances from cluster centroids

Given a library of surface patches, any surface patch may be marked with its distance to the closest library element (DCLE). The essence of the K-means algorithm is optimization of the average DCLE within the clusters. Thus, one may expect a low average of DCLE values for training set patches and higher values for unrelated patches. Figure 3 compares the distribution of training set DCLE values with six test set distributions: that of the native structures and those of the first, most confident, models submitted by five state-of-the-art CASP8 structure prediction servers. The DCLE distribution of patches extracted from native test set structures is almost indistinguishable from the training set distribution, which indicates that the library is not over-fitted. On the other hand, The DCLE distributions of the decoy patches, are significantly wider (Wilcoxon rank sum test, *p*< 10^-30^), with larger averages. This difference is large enough to distinguish native structures from a set of five decoy structures in 68% of the test set proteins (Table 1). The random expectation is 1/6, i.e., 16.6(± 7.7)% (where the standard deviation of 7.7 was estimated by 10,000 bootstrap re-sampling iterations).

**Table 1:** Ranking the native structures among 6 conformations (native and five predictions by servers)

Rank^a^	1	2	3	4	5	6
% Ranked ± std^b^	**74 ± 7**	14 ± 7	3 ± 7	4.5 ± 7	4.5 ± 7	0 ± 7

^a ^Best fitting structure to the patch library is ranked first

^b ^The random expected value is 16.6%

While compatibility with the surface patch library discriminates between native structures and decoys, it provides a weaker clue regarding the quality of the decoys themselves. The best decoys (by RMSD), are only slightly enriched within the most compatible decoys (Tables 2 and 3), probably because on average the decoys are more similar to one another than to the native structure. Decoy quality assessment by GDT_TS resulted in similar results (data not shown).

**Table 2:** Ranking the model with the best RMS score (over all residues) among the top models generated by the five prediction servers

Rank^a^	1	2	3	4	5
% Ranked ± std ^b^	**29 ± 7**	20 ± 7	13 ± 7	20 ± 7	18 ± 7

^a ^Best fitting model to the patch library is ranked first

^b ^The random expected value is 20%

**Table 3:** Ranking the model with best RMS score (over all residues) among five models generated by the same server (CASP8 servers 425 and 426)

Rank^a^	1	2	3	4	5
Server 425: % Ranked ± std ^b^	**28 ± 7**	17 ± 7	25 ± 7	7 ± 7	23 ± 7
Server 426: % Ranked ± std ^b^	**23 ± 6**	25 ± 6	15 ± 6	14 ± 6	23 ± 6

^a ^Best fitting model to the patch library is ranked first

^b ^The random expected value is 20%

### The relative size of clusters

**Figure 4:**
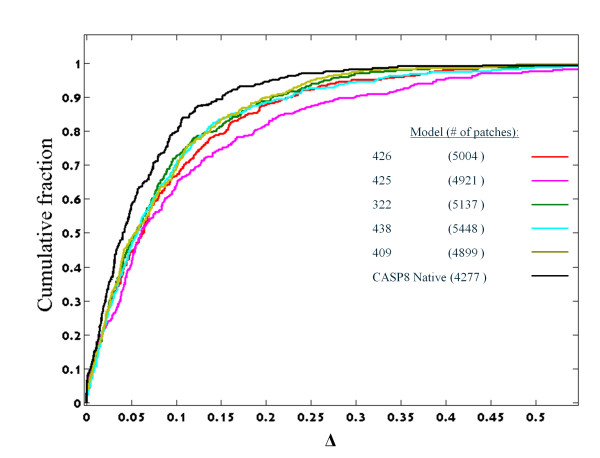
**Relative cluster preferences (Δ ) of patch sets are represented by cumulative distributions**. A bias towards low Δ values indicates that the cluster preference of the set of patches is similar to that of the training set. The native structures of the test set are significantly more similar to the training set than the servers' sets (p < 10^-4 ^by Wilcoxon rank sum test). Note also the considerable differences between the preferences of the various servers.

Cluster preference is another property that distinguishes between the patches of native and decoy structures. Formally, for a set of patches *Q *(e.g., patches extracted from some decoy set) this preference is a vector *F*(*Q*) = { *f*(*Q*,*C*_1_) .... f(*Q,C*_k_) }, where *f*(*Q,C*) is the fraction of *Q *elements that are closest to the centroid of cluster *C*, and *k *is the number of clusters. Figure 4 presents a cumulative distribution of Δ_i _= | *f*(*Q*^0^,*C*_i_)- *f*(*Q,C*_i_)|, per each data set Q, where *Q*^0 ^is the set of training patches. The Δ values of the test set native structures are significantly lower than those of the decoys (*p*< 10^-4 ^by Wilcoxon rank-sum test), indicating that the native structure preferences are far more similar to those of the training set than the preferences of the decoys. Curiously, not only do the native structures differ significantly from the decoys, the server structures differ considerably among themselves.

## Discussion and Conclusions

This work presents a new library of surface patches analogous to the fragment libraries that had a considerable impact on computational structural biology over the last twenty years [23]. Here, to demonstrate the significance of our library, we use it to compare patches taken from native structures and from decoys generated by state-of-the-art protein structure prediction servers. Our results show that the clusters are meaningful, and capture genuine aspects of native protein surfaces. Specifically, patches of decoys generated by servers are significantly different from patches of native proteins. Furthermore, this difference has a predictive power allowing us to identify native protein structures within a set of server models.

This phenomenon can be only partially attributed to the qualities of the models as measured by the standard RMSD and GDT_TS scores. Patch-derived measures (e.g., DCLE) are not correlated with RMSD or GDT_TS (data not shown), Good models (e.g., of low RMSD) are as prone to non-native surface patches as bad ones. Thus, we cannot use it to reliably rank decoys. On the other hand, we hope that our library will shed light on inherent limitations of the current modeling techniques. Such limitations in the representation of surfaces may be overlooked by the current model assessment procedures. However, they may drastically reduce the applicability of models for real life problems that often involve surface interactions. The characterization of these discrepancies between model surfaces and the surfaces of native structures is an obvious direction to continue this study. We hope that it would lead to some insight about the limitations of current modeling procedures and eventually to better model building techniques. A few other future applications are listed below.

Our approach to surface patch sampling requires quite a few parameters, such as the patch radius and the number of clusters. Due to the exploratory nature of the current study, we have decided to avoid a time consuming systematic search for the optimal values of these parameters. Some of them were assigned arbitrary values, and for others we sparsely sampled a wide range of values (data not shown). Although some values generated better results than others, the results were qualitatively similar, suggesting that the approach presented here is stable and viable.

Protein structures are extremely complex entities and no single perspective exposes all their properties. In the past, new protein representations (e.g., fragments [23], and rotamers) opened the way to diverse lines of study. One may speculate a similar trend here. Possible directions include functional inference from patch content, evolutionary conservation, and diversification of patch content and graphical representation of protein surfaces with patches as nodes and patch overlap as edges. The latter suggests new directions for structure-based comparison, search, and classification.

## Methods

### Data Sets

The training set, which is available online at http://www.cs.bgu.ac.il/~keasar/patchLibrary/domain_names.html, is the one previously used by Kolodny et al. [25] and includes 200 unique domains from SCOP version 1.57. These domains were solved using X-ray crystallography at high resolution [31] and each of them has the highest ranking SPACI scores [32] in its SCOP category.

The test set includes both native structures and their server-predicted models (decoys). These structures correspond to 55 CASP8 [33] single domain targets that were solved by X-ray crystallography and are non-homologous to the training set proteins. Specifically, the training set proteins have a BLAST [34] E-value of at least 10^-3 ^when run against the training set. The decoys were generated by five top CASP8 servers (Table 4), and are available through the CASP8 web site. Following the CASP regulations, each server submitted five models per target, ordered by confidence.

**Table 4:** Decoy data sets from CASP8

Server name	Server Group	Number of Models	Number of Patches
Zhang-Server	Zhang	426	5004
Baker-ROBETTA	Baker	425	4921
Phyre_de_novo	Sternberg	322	5137
RAPTOR	Gao	438	5448
pro-sp3-TASSER	Skolnick	409	4899
Native^a^			4277

^a^There are 55 native structures.

### Identification of surface atoms

We consider an atom of type *t *(e.g., Alanine-Cα) to reside on the surface if its accessible surface area, calculated by PROGEOM [35], is at least *α*.access_surf_*t *_(Figure 1a). Here, access_surf_*t *_is the 99th percentile of the cumulative distribution of accessible surface area within all the atoms of type *t*, and *α *= 0.9. The empirical adjustment of these two parameters reduces the effect of errors in the crystallographic data (e.g., missing side chains that superficially expose backbone atoms), and ensures continuous coverage of protein surfaces.

### Patch definition

We define surface patches as sets of surface atoms centered about all solvent exposed β-carbons, which we denote pivots (Figure 1). Each patch includes the central pivot and all surface atoms within a given radius around it. This radius is a critical parameter as the number of atoms within a patch is strongly dependent on it. Thus, a large radius results in large numbers of atoms and long evaluation times for the combinatorial distance measure (see below). On the other hand a too small radius may leave surface regions uncovered. A preliminary study suggested 7Å as a reasonable compromise that keeps a manageable number of atoms in a patch (around 25 on average) and provides a continuous coverage of proteins' surfaces by overlapping patches.

### Measuring the distance between two patches

Given two patches *A *and *B*, we look for an optimal superposition in terms of structure and chemical properties, and define the distance between *A *and *B *as the minimal RMSD under a set of chemical constraints. If the compositions (see below) of the patches are too remote to allow meaningful superposition, we set the distance to infinity.

More formally: Let the patches be the respective sets of atoms in *A *and *B, A *= {*a*_1_,...,*a_n _*and *B *= {*b*_1_,...,*b_m_*. Let *T_iA _*be the number of atoms of type *T_i _*in patch *A *and *rg*(*A*) the radius of gyration of *A *(symmetrically for *B*).

Notice that , and .

The patches *A *and *B *are compatible if

,, and 

The threshold values for size difference, chemical difference, and radius of gyration difference were arbitrarily set to Φ_1 _= Φ_2 _= 0.2, and Φ_3 _= 5Å. The distance between incompatible patches is infinite.

Let *t*: {set of all atoms} → *T *be a mapping so that for an atom *a, t*(*a*) is the atom's type. A mapping *f*, from *A *to *B*, is **proper **if it satisfies *f*(*a*) = *b *if and only if *f*(*b*) = *a *and *t*(*a*) = *t*(b).

Let *F *= {*f*_1_,..., *f*_k_} be the set of all proper mappings of *A *and *B*.

Then, the distance between *A *and *B *is:

where RMSD(*A,B,f*) is the optimal superposition [14] of the atoms of *A *and *B *that are mapped by *f*.

**Figure 5:**
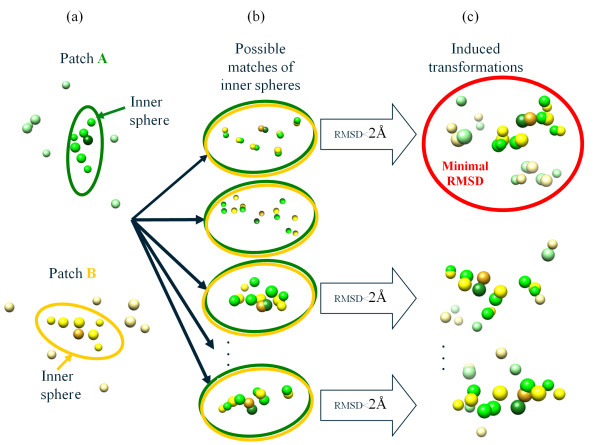
**The distance approximation heuristic**. (a) Given two patches *A *(in green) and *B *(in yellow), we consider their inner spheres (pivots are marked using a darker shade). (b) Then, we enumerate all possible matches of the inner spheres. (c) If the RMSD between the inner spheres is less than 2Å, it serves as a seed for mapping the full patches. Finally we pick the superposition that yields the minimal RMSD value (circled in red).

In practice, finding the optimal mapping is a hard combinatorial optimization problem, although the requirement for compatibility provides a filter that reduces the number of these calculations considerably. Thus, the use of the exact distance definition above might have rendered the calculation of numerous distances infeasible. Instead, we use a heuristic approximation that reduces the number of tested mappings. To this end, we define the inner sphere of a patch to be a sphere, centered at the pivot, of radius *r *< 7Å, which is adjusted so that the number of surface atoms in the inner sphere is between 4 and 9 (see Figure 5a). We then exhaustively enumerate all possible chemically valid mappings between the inner sphere of one patch and the inner sphere of the other patch (Figure 5b). The RMSD between these inner spheres is measured after optimal least-squares superposition. If this RMSD is less than 2Å, the transformation it implies serves as a seed for matching the full patches *A *and *B*. If no seed was found, the distance between the patches is taken to be infinity. Once the transformation of a seed match was applied to the full patches, we match the atoms of *A *and *B*: each atom of *A *is matched according to proximity and chemical attributes to the best fitting atom in *B *(Figure 5c). Now we have a mapping between *A *and *B *for each seed. For each such mapping we compute the RMSD between *A *and *B *and pick the matching with the lowest RMSD.

### Outlier weeding

Patches that are distant from the majority of other patches are outliers; we weed them out in a pre-processing step to avoid numerous non-informative singleton clusters. Here, we define an outlier as a patch that has a distance greater than 2.5Å to more than 90% of the other patches; this filters out 1.51% of the surface patches. A closer look at some of the outliers reveals a diverse population. Some of them are unique (within our dataset) functional elements like metal binding sites, for example the small protein 1VFY contributes four outliers due to its two metal binding sites and a large fraction of unstructured chain. Others are artifacts of using domains instead of whole proteins, for example 1JHG, which is a homo-dimmer, contributes five outliers. Three of them are actually buried by the other subunit. Finally, some of the outliers do not show any peculiarity that we could identify. Their uniqueness may be simply an artifact of the relatively small size of our dataset.

## Authors' contributions

RG participated in the project design, wrote software, generated the data and analyzed it, and drafted the manuscript. RK participated in the project design and supervised its clustering aspects. KK participated in the project design and supervised its computational geometry aspects. CK conceived the project and coordinated it. All authors took part in manuscript writing, read the final version and approved it.
